# Systems biology of lactic acid bacteria: a critical review

**DOI:** 10.1186/1475-2859-10-S1-S11

**Published:** 2011-08-30

**Authors:** Bas Teusink, Herwig Bachmann, Douwe Molenaar

**Affiliations:** 1 Systems Bioinformatics / NISB, Faculty of Earth and Life Sciences, VU University Amsterdam, De Boelelaan 1085, 1081 HV Amsterdam, The Netherlands; 2 Kluyver Center for Genomics of Industrial Fermentations / NCSB, Julianalaan 67, 2628 BC Delft, The Netherlands

## Abstract

Understanding the properties of a system as emerging from the interaction of well described parts is the most important goal of Systems Biology. Although in the practice of Lactic Acid Bacteria (LAB) physiology we most often think of the parts as the proteins and metabolites, a wider interpretation of what a part is can be useful. For example, different strains or species can be the parts of a community, or we could study only the chemical reactions as the parts of metabolism (and forgetting about the enzymes that catalyze them), as is done in flux balance analysis. As long as we have some understanding of the properties of these parts, we can investigate whether their interaction leads to novel or unanticipated behaviour of the system that they constitute.

There has been a tendency in the Systems Biology community to think that the collection and integration of data should continue ad infinitum, or that we will otherwise not be able to understand the systems that we study in their details. However, it may sometimes be useful to take a step back and consider whether the knowledge that we already have may not explain the system behaviour that we find so intriguing. Reasoning about systems can be difficult, and may require the application of mathematical techniques. The reward is sometimes the realization of unexpected conclusions, or in the worst case, that we still do not know enough details of the parts, or of the interactions between them.

We will discuss a number of cases, with a focus on LAB-related work, where a typical systems approach has brought new knowledge or perspective, often counterintuitive, and clashing with conclusions from simpler approaches. Also novel types of testable hypotheses may be generated by the systems approach, which we will illustrate. Finally we will give an outlook on the fields of research where the systems approach may point the way for the near future.

## Review

### Introduction

Systems biology is a relatively new field of science that employs, in an iterative fashion, a combination of quantitative data, mathematical modeling and theory to come to a “systems-level” understanding. We interpret this as an understanding how the behaviour of the system, be it the frequency of a microorganism in a microbial community, or the flux through a metabolic pathway, depends on the properties of the components of the system, and the interactions between the components. It is therefore not the opposite of reductionism: in its bottom-up manifestation, systems biology uses the reductionist’s data (properties of the components), and builds a picture of the predicted collective behaviour if the interactions are included. In its top-down manifestation, systems biology aims at component and interaction identification from large data (omics) sets, where it has strong connections with (and may even be indistinguishable from) bioinformatics.

Systems biology has penetrated mainstream biology considerably [1]. Also in the field of lactic acid bacteria research, the systems biology approaches has quite a tradition. In this review, we want to illustrate what systems biology has brought the LAB field, through a number of selected cases. This review has a stronger -but not exclusive- focus on the bottom-up approach, and on microbial physiology, in particular metabolism. We will start with genome-scale metabolic models and their approaches, which may be considered a compromise between bottom-up and top-down systems biology. Then, after identifying specific limitations in these type of models, we will focus on kinetic models of LAB physiology, to discuss the "effective cause" (the how) and the "final cause" (the why) of regulation of metabolism in LAB. Finally, we will scale up and consider cells as components in a community of cells and discuss metabolic regulation strategies within the framework of population dynamics. We will end with some perspective of what we believe are some of the dominant future developments in the systems biology field, relevant for LAB research.

### Genome-scale metabolic models

Today’s interest in systems biology is largely fuelled by high-throughput techniques that generate large amounts of data. There is a general consensus that functional genomics has enormous potential in the life sciences, in particular in biotechnology and medicine. How to use these technologies most efficiently, either for fundamental understanding, biomarker discovery or concrete biotech applications, is an area of active research. It is clear that the volume and complexity of the data are becoming too large to cope with by biologists alone, especially when the latter are poorly trained in advanced mathematics and computation (which is unfortunately still largely the case). So there is an understandable need from the biologist’s perspective for help in mining, interpreting and using the datasets that they collect. Such activities require modelling of one form or the other [2].

Biostatistics and bioinformatics offer help in the analysis of genome-scale data sets, but they often rely on purely mathematical and statistical analysis [3]. Although extremely useful, it ignores what is often referred to as “legacy data”, i.e. the large body of biological knowledge that is often scattered in literature and therefore poorly accessible. Moreover, many of the techniques were not designed to incorporate *a priori* knowledge, even if it is available [3]. “Bottom-up” systems biologists, on the other hand, construct detailed mechanistic models that aim at a fundamental understanding of systems behaviour [1] (see also the section on control of primary metabolism of LAB).

Using genome-scale reconstructions, and their corresponding models, may be considered as a “middle-out” approach, since they combine -omics data with more traditional modelling strategies. All aspects of genome-scale metabolic models have been extensively reviewed in recent years [4-10]. In this section we will describe some of its application to metabolic networks of LAB. These applications can be divided into three main application areas: (i) advanced bioinformatics and data analysis; (ii) quantitative analysis and prediction of fermentation; and (iii) exploration and discovery of metabolic potential.

#### Advanced bioinformatics and data analysis

A genome-scale metabolic model, or metabolic reconstruction, is nothing more and nothing less than a manually curated inventory of all gene-protein-metabolic reaction associations of an organism [5,11]. It is based on a combination of bioinformatic inference of gene function, experimental evidence in the form of biochemical studies and physiology (e.g. auxotrophies for amino acids or vitamins [12]), and literature searches. For information on how to make such models, we refer to some reviews on this topic [4,5,11]. Quite a few genome-scale metabolic models for LAB are available [13-15]. Once the often complex and many-to-many gene-protein-reaction relationships are mapped out, these same relationships can be used for integration of data sets that refer to these network constituents. In general, a genome-scale metabolic reconstruction provides what Palsson called a "context-for-content" [16], and such pathway analysis has been used in numerous studies, ranging from metabolic interpretation of fitness screens or knockouts [17-19], functional association studies [20], and studies on the evolution of genomes [21] and metabolic networks [22,23]. A relatively simple example in LAB research was the use of metabolic maps to plot microarray data. This analysis was used by Stevens et al [24] to identify CO_2_ as a potential cause for growth retardation in aerated cultures of *L. plantarum*. A more formal and statistical method, with exactly this aim, was developed by J. Nielsen's group, called *reporter metabolite* analysis [25].

#### Quantitative analysis and prediction of fermentation

Genome-scale metabolic models are stoichiometric models, and hence can be used to analyse (and sometimes predict) fluxes in metabolic networks, an area often referred to as metabolic flux analysis (MFA, see for an excellent review on different modelling techniques in metabolic engineering [26]). In that field, focus has mostly been on estimating the internal fluxes from external fluxes (consumption and production rates), and ^13^C-label incorporation in metabolic pools [27]. Models were developed specifically for precise flux estimations [28,29], but genome-scale metabolic models turn out to have many more degrees of freedom than the traditional stoichiometric models used for flux analysis (think in the order of one hundred degrees of freedom, see e.g. [15]). This is primarily caused by lumping and simplifications in the latter case: when the model is constructed based on the genome, many additional catabolic pathways and sugar uptake pathways are included that would be irrelevant for dedicated MFA models.

When uptake and production data are available, an extremely useful approach is to set these data as flux constraints in the model, and then perform Flux Balance Analysis (FBA). FBA requires an objective (biomass production rate, or ATP production rate) that is optimized [9]. The optimisation algorithm, called linear programming, will search for flux distributions that will maximize the objective function. Importantly, the solution space, *i.e.* the space of all feasible (not necessarily optimal) flux distributions, is bounded in this case by the measured uptake and production rates. Hence, the algorithm will find optima at such boundaries. An interesting measure produced by the linear programming algorithm is the so-called reduced cost: it quantifies how much the objective would increase (or decrease in case of a minimization problem) if the boundary was allowed to be stretched a little [30]. If this reduced cost is non-zero for a measured uptake flux, this indicates that the uptake of this compound potentially limits the objective function [15].

There is one technical detail that is important however: although FBA is guaranteed to find the global optimal value for the objective function, there could potentially be many different flux distributions that provide that value. Thus, FBA solutions are not unique [31]. One should therefore test which reactions have a unique value in the optimum, and which ones are still free to vary. This can be tested with flux variability analysis (FVA), which minimizes and maximizes each flux in the network in the optimum [32]. FVA on models of LAB constrained by experimental data actually result in FBA solutions that are quite unique, and most variability is only minor and not affecting the reduced cost analysis. This is only the case if the network is energy-limited and ATP production and consumption by biomass formation are strictly coupled; releasing this constraint result in much more flexibility, e.g. in futile cycles [15]. Our colleagues found the same for *E. coli* models (Bruggeman, Kelk, Olivier and Stougie, unpublished results), so this is not specific for LAB.

In two studies in *L. plantarum*, interesting new biological discoveries were made applying reduced cost analysis. In a study by us, it was found that the catabolism of branched chain and aromatic amino acids contributed to ATP production [15]. Detailed analysis showed that the catabolism of these amino acids constitute a transhydrogenase activity that could replace the conventional NADPH production by the oxidative part of the pentose phosphate pathway. Under anaerobic conditions, this is beneficial as it removes excess NADH and converts it in NADPH required for fatty acid biosynthesis. This transhydrogenase activity, hidden in the metabolic network, was also found in *Streptococcus thermophilus*, which lacks the oxidative steps of the PPP, and in this organism’s amino acid catabolism could be a major source for NADPH [14], although there is also a NADP-dependent GAPDH enzyme present that could fulfil this role. In *S. pyogenes* (and many more streptococci), a similar situation appears present (Levering, unpublished results).

In the second case, reduced costs analysis was used to understand enigmatic production of amino acids at “zero growth” in *L. plantarum*[33]. In this study, retentostat cultivation was used to grow *L. plantarum* at progressively lower growth rates, and increasingly more amino acids were secreted (such as aspartate and arginine). Interestingly, other amino acids, notably aromatic and branched chain amino acids, were taken up in excess. Microarray data showed upregulation of plant-specific gene clusters [34]. Since the catabolic products of branched-chain and aromatic amino acids are identical or similar to known plant-hormones, these data suggest that *L. plantarum* behaves as if in a plant-like environment [33]. Reduced costs showed that under these conditions, amino acids were secreted as an alternative means to export the excess nitrogen arising from branched-chain and aromatic amino acid catabolism.

#### Exploration and discovery of metabolic potential

Thus, genome-scale models can be used to analyse complex uptake and production data to get insight in limitations of fermentations and growth, with applications in growth medium optimisation. Moreover, as they constitute a comprehensive inventory of the metabolic potential of an organism, genome-scale metabolic models, in contrast to traditional MFA models, can lead to new pathway discoveries, as illustrated above with the transhydrogenase example. One more example of this is a putative transketolase cycle in *L. plantarum*, which could result in an aerobic “combustion” of glucose to CO_2_ and H_2_O, with a stunning 6 moles of ATP per mole of glucose as the result [35]. Although this cycle appears not to be operative, it is an interesting “hidden” pathway that may be worth exploring further.

Another option is to use genome-scale models for what-if scenarios, which would be very useful for metabolic engineering and synthetic biology. New pathways can be introduced *in silico*, and tested whether all co-factors can be made in the right proportions; the maximal theoretical yield can be computed, which by-products will be formed, and so forth [36]. Conversely, gene deletions and/or combinations thereof can be scanned to find scenario’s that would increase the flux towards the product of interest [37-39]. Although this approach has been demonstrated to work in *E coli*, notably by the group of S. Y. Lee [40,41], not many examples are found for LAB. This is likely to be caused by the still relatively poor penetration of systems biology in this traditional field, and because of its association with food, which obviously hampers metabolic engineering and synthetic biology approaches. We found one example where FBA was used to model protein over-expression in *L. lactis*[42].

#### Limitations of genome-scale models

Finally, we would like to remind the reader of the fact that genome-scale metabolic models are stoichiometric models that lack any kinetic detail. Hence, only ratios of fluxes can be computed, i.e. only questions such as “how much comes out if I put in so much?” can be addressed. These are yields, and stoichiometric models can only predict yields, or in the case of FBA optimal yields. In the section on optimality, we will revisit this point and argue that organisms, and LAB in particular, very often are not selected for yield or efficiency, but for rate. Therefore all predictions with genome-scale metabolic models will have to be weighted against this potential confounder (see [43] for examples). The scenarios should be viewed as hypotheses in guiding the next experiments, and in that sense the models are very useful, despite their limitations.

### How is primary metabolism in LAB controlled (regulated?)

Mechanistic explanations of metabolic behaviour and how this is manipulated by the organism itself, or can be manipulated through metabolic engineering, are at the heart of much of the research on LAB. A number of mathematical modelling approaches have been used in LAB research to integrate experimental data from detailed biochemical studies on transport process [44] and enzyme kinetics (see, e.g. [45,46]), supplemented with flux and metabolites measurements, such as in-vivo NMR [47,48]. The genomics approach to LAB physiology has been accompanied in recent years by the use of genome-scale metabolic network models [15], as discussed above. Here, we want to discuss several attempts to model LAB metabolism dynamically, with the use of kinetic (differential-equation based) models, of which several have been published. The focus will be on the assumptions and limitations of these models, and to what extent they have helped to understand the wealth of physiological data, or to generate hyptheses.

**Figure 1:**
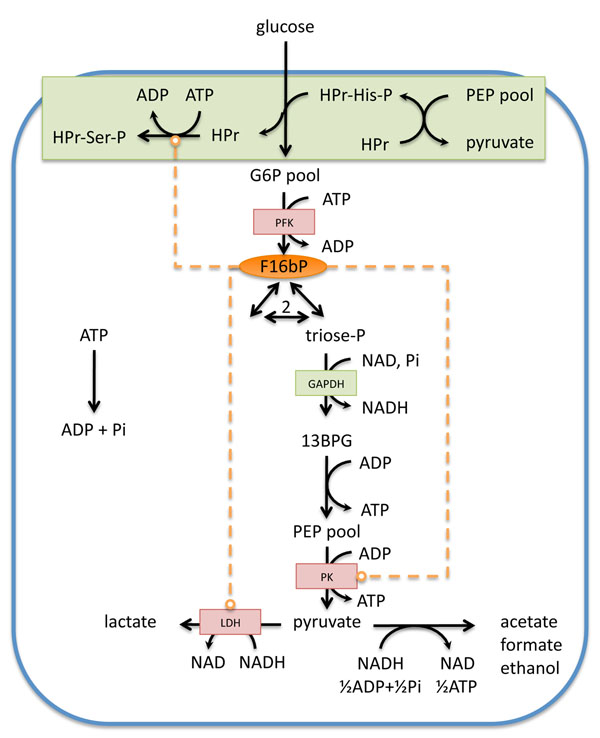
Primary metabolism of *L. lactis* with major players discussed in the main text. Indication of the (positive) regulatory feedback and feedforward loops that involve F16bP in dashed orange line. In red are the enzymes of the *las*-operon. In green boxes the PTS system and GAPDH, respectively. G6P and PEP pool indicate pools of intermediates that are considered in rapid equilibrium.

Not surprisingly, kinetic modelling efforts in *L. lactis* have been almost exclusively focused on glycolysis (see Figure 1 for main features of the pathway relevant for the discussion). There have been two main approaches to model glycolysis in *L. lactis*. The first flavour are variants of the “Hoefnagel” model [52-54], constructed in the "*in silico*-cell” spirit, which means that all parameters in the model are based on enzyme kinetics obtained *in vitro*[55]. The parameterised rate equations for all the enzymes are then put together to compute the behaviour of the whole pathway, in terms of metabolite levels and fluxes. No fitting is involved; data and model predictions are being compared to identify mainly structural anomalies in the model. The second approach is based on fitting time-series data of metabolites, almost exclusively *in vivo* NMR data from the group of H. Santos [56,57]. In the latter approach, no biochemically realistic rate equations are used, but approximated kinetics (in the power-law format) with less parameters and mathematical attributes that make them easier to handle, notably by the fact that the differential equations have analytical solutions [58]. Recently, a third approach was presented and compared to the power-law modelling: this approach, based on so-called dynamic budget theory, is working at a much higher abstraction level [59]. The study bears similarities to the economic perspective of cell growth presented by us [60], but it has less biochemical detail than the two main approaches discussed here, and therefore does not help in explaining mechanisms, or identifying potential targets for metabolic engineering.

Both approaches have their pros and cons. The Hoefnagel approach is closer to biochemistry and biological intuition, but suffers from insufficient kinetic data (e.g. many enzyme kinetics were included from other organisms than *L. lactis*), and the potential *in vivo* – *in vitro* differences that are unavoidable and difficult to address. Moreover, the enzyme kinetics are usually taken from databases such as Brenda or Sabio-RK [61], in which the assay conditions for each enzymes is likely to be different. This touches upon an important standardisation and documentation issue that we will discuss at the end of this section. These limitations to *in vitro* kinetics result in parameter values that are rather uncertain, yet no comprehensive (global) sensitivity analysis has been presented as far as we know that addresses which parameters in the model have a large effect on the dynamics and control of glycolysis. Given these limitations, the correspondence between the models and experimental data are surprisingly good, and they form a solid basis for further refinement of the models, and of understanding the biochemical basis of primary metabolism in *L. lactis*.

The data-fitting, or inverse-engineering, approach as practiced in particular by Voit and colleagues [56,57], has a thermodynamic underpinning [56,58] but has the disadvantage that there is no clear mapping to biochemistry. The simplifications allow for an analytical solution of the ordinary differential equations that make up most kinetic models. This is potentially a great advantage to obtain deeper insight into how a particular design or set of parameters affect pathway behaviour. A recent example in *L. lactis* exploits this using a similar approach, not with power-law kinetics but with linlog kinetics which also renders analytical solutions to the set of mass balance equations [62].

The main finding, as far as we are aware, resulting from the inverse-engineering analysis is the importance of the feed-forward activation of F16bP on PK. This feed-forward loop is allegedly required for the rapid increase in PEP upon glucose exhaustion [57]. Since PEP is the substrate for the PTS system that takes up glucose, the increase in PEP has been rationalized as a strategy to ensure prompt uptake of glucose once available again. In fact, Hoefnagel *et al* already made a similar observation in their *in-silicon* cell model [53]. They also proposed that F16bP activation, but also inorganic phosphate (Pi) effects on PK and regulation of PFK by PEP are important for an increase in PEP and slow depletion of F16bP.

Backed up by on experimental evidence Hoefnagel [53] stressed the importance of Pi as a free variable, rather than an input variable used by Voit [57]. However, he did not mention what we think is the most important consequence of considering Pi as a free variable: it renders the total pool of phosphate in the cell as a conserved pool. This is caused by what is called moiety conservation [63]: since there is no net transport of phosphate in the models (glucose comes in, and acids go out), and cells cannot make phosphate *de novo* (unless –unexpectedly- they would be capable of nuclear reactions), the total amount of phosphate contained in all metabolites such as PEP, F16bP, ATP and Pi, cannot change (other moiety conservations are for example the total pool of Coenzyme A, or the sum of NADH and NAD). Note that these moiety conservations may be artefacts of models since we ignore potential sources an sinks, such as polyphosphate, but these fluxes are likely slow compared to the high glycolytic flux. Hence, if the kinetics in the model are such that F16bP drops because of glucose exhaustion, the associated phosphates *ha*ve to go to some other pools, notably PEP and Pi. So the question really is what the most important kinetic parameters are that cause the change in distribution of phosphate over the glycolytic pools, ATP and Pi. This has not been fully addressed yet, and thus the claim that the feed-forward activation of PK by F16bP is relevant in this respect, is in our opinion still pending.

It should be noted moreover, that the F16bP feed-forward loop on PK is by no means unique for *L. lactis*. Most if not all glycolytic pathways exhibit this (over 30 cases in the Brenda database), notably organisms that do not have a PTS glucose uptake system, and hence, do not rely on high PEP levels to “start-up” glycolysis upon glucose re-addition. So, one may wonder about the functional interpretation of the feed-forward activation in other organisms. One hypothesis we have is linked to the kinetics and thermodynamics of GAPDH: this enzyme operates near equilibrium and is very sensitive to mass action [64]. High F16bP levels likely indicate high flux (as in *E coli*[65]) and hence signal that a high(er) activity of GAPDH is required. The feed-forward loop on PK should help in pulling at the metabolites in lower glycolysis, hence reducing the products of GAPDH. The latter scenario fits with the inhibitor study of GAPDH [66], showing that the activity of GAPDH exerts high control on the glycolytic flux. We would like to note that this result is actually not in conflict with the study from P.R. Jensen’s group in which they showed that varying the expression of GAPDH did not affect the glycolytic flux [67]: these studies do not strictly measure the metabolic control coefficient, as explained in the optimality section.

Apart from the feed-forward loop, there is another important regulatory loop in the glycolytic system in *L. lactis* that has not received the attention we think it deserves: the negative feedback of F16bP (and Pi) on the PTS system. Within the PTS system in gram positives, the HPr protein has a dual role: when phosphorylated at the His-15 residue, it allows phosphate group-transfer within the chain of phosphorylation events that lead to uptake and phosphorylation of glucose. When phosphorylated at the Ser-46 residue, however, HPr acts as a signalling intermediate in glucose repression, activating CcpA [68]. The latter state of HPr is promoted by F16bP and is not available in the transport process, thus constituting a F16bP-mediated negative feedback on glucose uptake [69]. Studies in yeast, and a comparative analysis of glycolytic designs, strongly suggest that this feedback is essential for robustness against sudden changes in glucose availability [69]. This feedback should also be relevant in evaluating the potential effects of PEP on the PTS system and restarting glycolysis: modelling efforts [54] looking at the effect of pH suggested that this effect of PEP could explain the negative effect of low pH on glycolytic flux, but this was assessed without taking the potentially counteracting effect of F16bP into account (as F16bP was also lower at the lower pH, but not taking into account [54]).

Finally, one of the more complex behaviours in *L. lactis* glycolysis that is screaming for a mechanistic explanation –still- is the (often gradual) “switch” between homolactic and mixed acid fermentation, i.e. the fact that *L. lactis* exhibits mixed acid fermentation (acetate, ethanol and formate as major products) at low dilution rates in the chemostat, and homolactic fermentation at high dilution rates (see [70] for a clear example). The “final cause” of this switch will be discussed in the next optimality section, but the mechanism of the switch (“efficient cause”) has also been widely debated in the literature, as reviewed earlier [48]. Taking a systems perspective, there are two points to make.

One, it is essential to be precise and make a distinction between control and regulation, as this has confused the literature quite a bit. Within Metabolic Control Analysis, control means the ability of an enzyme activity to change a flux or concentration; regulation is the way in which the flux or concentration is actually altered [refs]. For example, in *L. plantarum* ATP demand has some control on flux [72]: this means that with increasing the ATP demand rate, the glycolytic flux increases. *How* this change is brought about, is the realm of regulation: it depends on how the regulatory network “plays the control knobs in metabolism”. So if one states that “the NADH/NAD ratio determines the switch”, one probably means that reactions that affect the NADH/NAD ratio have control over the lactate to acetate flux ratio, *via* the NADH/NAD ratio. We feel the latter statement is much more precise and gives rise to less confusion, and better design of experiments to prove or disprove this. We feel that systems biology and its concomitant precise theoretical definitions have a lot to offer in this respect.

Second, the switch is most likely the result of a number of feedbacks and allosteric regulatory interactions that take place simultaneously, and so a model is warranted that can quantify the contributions of the different mechanisms. This impact is not unlikely to change with conditions, and so the question “what causes the switch?” is most likely the wrong question to ask. We are developing a kinetic model that takes *in vitro* kinetics as the basis, and uses experimental time-series data to update or fit parameters. Subsequently, global parameter sensitivity analysis should be applied to assess their impact in the light of their uncertainty, and to assess which parameters affect flux, and the switch. We think that this is the best approach to tackle this important physiological observation.

### Optimality as an explanation of regulation

The distinction made between different types of explanatory causes by Aristotle is still applicable in biology. In particular, it is important to distinguish the efficient and final causes (*causa efficiens* and *causa finalis*) in modern molecular biology. Efficient causes are, for example, the molecular details of passive or active regulatory mechanisms that lead to a certain behaviour. The apparent final cause, or function of such regulatory mechanisms is the efficient survival and replication of organisms in which they act. Darwin already noticed that this final cause, as he also calls it, is programmed by the mechanism of natural selection [73]. Therefore, when details of regulatory mechanisms have been revealed by molecular biological research, the scientific quest is not finished. There is still the relevant question whether this mechanism effectively serves survival and replication. And by “effective” we mean in a manner that is near to optimal given the tools that the organism has at its disposal, since the second consequence of natural selection is that the fitness of the organism to serve survival and replication will be improved until some kind of maximal use of available resources is reached. The answer to this question is often not so easy to give, because of the complicated interactions of components within the organism and between the organism and its environment, as well as the obscurity of how exactly fitness, or success in replication and survival is determined in the often dynamic biotic and abiotic environment. Systems biology can play a role in understanding the relation between the efficient and final causes, as we will try to illustrate. The sort of models used in such research is typically not of the (once hailed) comprehensive “*in silico* cell” type [55], but much more basic and easier to understand, though often yielding surprising results.

We may expect that optimal use of available resources is easiest to understand for organisms that replicate in relatively simple environments, of constant and homogeneous nature and with competitors of their own kin only. The microbiologist immediately thinks of a pure culture in the chemostat or propagated by serial transfer in a constant medium. It has been shown that it is possible to understand aspects of central metabolism in organisms selected under such conditions from the perspective of optimal use of resources. A nice example was given by Ibarra *et al.*[74] where it was shown that during serial transfer *E. coli* adapts through mutations to growth on glycerol, and that the mutants converged to a metabolic profile that could be predicted from optimality principles using FBA. In this case optimality of fitness was assumed to correspond to a maximal biomass synthesis rate at a limited glycerol uptake rate. Hence, it seemed that FBA was able to explain central metabolic profiles using assumptions about the final cause of metabolic regulation under the given condition, i.e. production of biomass. However, in the same paper it was shown that FBA failed to predict the metabolic profile of *E. coli* growing on glucose, in particular the production of acetate at high glucose concentrations. Similarly, FBA analysis on a genome scale model of *L. plantarum* predicted a mixed acid fermentation profile under all circumstances, instead of the experimentally observed lactate production [15]. Interestingly, also for *L. plantarum* adaptation to glycerol appeared predictable by FBA . The failure of FBA to predict adaptation under glucose conditions suggests that FBA perhaps lacks essential elements that are important for explaining optimal growth on abundant, energy-rich carbon sources. In fact, any energetically inefficient use of substrate, sometimes referred to as “overflow metabolism”, is predicted not to occur at any time by FBA, unless *ad hoc* capacity constraints on certain metabolic paths are imposed. This type of metabolism is, however, so abundantly observed in microorganisms [70,75-78] that it is hard to believe that it doesn’t result from fitness maximization.

Concentrating on LAB here, we see that a prominent feature of lactic acid bacteria is that they produce mainly lactic acid from sugars. In a number of cases like in *L. lactis*, mixed acid fermentation is observed at low substrate availability or during growth on sugars that lead to a low growth rate [70,75]. However, other species like *L. plantarum* display mixed acid fermentation only at extremely low substrate availability [15]. All this, despite the fact that mixed acid fermentation yields one additional ATP per glucose molecule. To the question why LAB produce lactic acid under certain conditions and mixed acids under other, there are answers in the literature stating that it is the NADH/NAD, ATP/ADP, or fructose-bisphosphate and triose phosphate concentrations or their ratio to free phosphate, as well as publications stating that it is the level of PFL or LDH that determines the choice [70,75,79-82]. But whatever the mechanism is by which the choice between efficient and inefficient metabolism is made, the question remains why lactic acid bacteria choose an energetically inefficient pathway anyway.

It seems as though the use of a mixed acid branch of fermentation has more disadvantages than an additional ATP could make up for. It is not immediately clear what these disadvantages could be. There are several hypotheses around to explain why LAB produce lactic acid despite having an energetically more efficient pathway at their disposal. One hypothesis states that this is a kind of chemical warfare, where other organisms competing for the same resources are inhibited in growth by high lactate concentrations. A similar hypothesis has been proposed to explain the production of alcohol by yeast [83]. The end products, alcohol or lactate, are produced as a collective effort by the population. The individuals pay for this warfare by the virtual loss of ATP that could have been gained in the mixed acid branch, in case of LAB, or in oxidative phosphorylation in case of yeast. The problem with this hypothesis is that one would expect that in pure cultures “cheater” mutants would arise that exclusively use the energy-efficient pathways. The implicit assumption in the warfare hypotheses is namely that such mutants would have a higher fitness than the wild type, because they use their substrate more efficiently. They would therefore take advantage of the warfare carried out by others without investing in it. When the need for chemical warfare disappears, as in single-species laboratory populations, the energy-efficient cheater mutants should even completely take over the population. However, there are no indications that such metabolic deserters exist in laboratory microbial populations.

Some time ago we published a hypothesis in which we proposed that several global characteristics of microorganisms, like overflow metabolism, might be the result of maximization of the growth rate [60]. For the hypothesis we assumed that the proper allocation of cellular resources determines the growth rate. The outcome of calculations on a self-replicator model showed that sometimes counterintuitive effects arise. The basic idea behind the model was that pathways generating additional ATP are generally longer, or they need more enzymes. So, in case of LAB, although additional ATP is gained from mixed acid fermentation, in comparison to homolactic fermentation at least five additional enzymes are needed to generate that ATP. Whether such an investment pays off depends on the environmental conditions, in particular on the substrate concentration, or more precisely the investment made to accumulate the substrate. The prediction of this model is that at high substrate concentrations faster growth is achievable with metabolically less efficient pathways. Furthermore, a clear shift in allocation of protein to the different branches is predicted, meaning that shift in use of the pathways should be accompanied by a shift in expression of the corresponding genes, because the investment in proteins of these pathways imposes a cost on fitness (see the discussion below under “*Signatures of optimality*”). Similar effects are predicted by models in the FBA framework when a crowding constraint is imposed on the total amount of enzymes [84]. Indeed there are observations that such shifts are accompanied by shifts in gene expression, for example in *E. coli*, *S. cerevisiae* and *B. subtilis*[77,78,85-87]. In chemostat experiments with *L. lactis* different observations were made. In *L. lactis* ML3 the specific activity of LDH increased with increasing growth rates and substrate concentrations, but the authors mentioned noticeable differences between strains in shift behaviour [70]. More recent experiments on *L. lactis* IL1403 where proteomics was used to measure relative protein concentrations in cells grown at different dilution rates showed that the level of LDH is relatively constant, but that protein ratios in the mixed acid branch decreased with increasing growth rate and substrate concentration [88,89]. Hence, although we cannot explain the constant activity of LDH (the fact that LDH is in the *las*-operon in *L. lactis*[90] does not help much, as it is not in the very homolactic *L. plantaru*m [91]), there are indications that the trade-off between investment in proteins of the mixed acid pathway and the benefit of additional ATP generation could play a role in determining the metabolic shift.

#### Signatures of optimality

Several publications can be found in the literature that show evidence of optimality of expression levels of proteins in microorganisms. For example, Dekel and Alon have shown in an evolutionary experiment that the level of β-galactosidase protein in *E. coli* quickly adapts to lactose concentrations in the medium. When cultures are serially transferred on media containing a fixed lactose concentration, mutants appear with an adapted expression of the *lacZ* gene [92]. An optimally tuned expression level is a compromise between the cost of expressing the β-galactosidase protein, which increases with increasing protein level, and the benefit of its activity which increases with increasing lactose concentrations. Dekel and Alon deduced cost and benefit of β-galactosidase levels at different lactose concentrations directly in terms of effects on growth rate. In the experimental setup used the growth rate was an important fitness component. The selection on maximal growth rates at the given lactose concentration and the trade-off between the cost and benefit on growth rate gave rise to selection of mutants with an increasing expression level of *lacZ* at higher lactose concentrations in the medium. Stabilization of expression levels occurred after 300-500 generations. This example showed that selection pressure on optimal expression of one protein, amounting to perhaps 3% of the total protein can be very strong (10000 tetramers per cell, 116 KDa per subunit, and a cell growing at a doubling time of 40 min. contains 235 fg of protein, Bionumbers 102019 and 104879 [93]).

A series of experiments from the group of P.R. Jensen at the Technical University of Denmark, show a signature of optimal expression levels in *L. lactis* and *E. coli* wild types. For example, when the growth rate of *E. coli* is measured at various expression levels of the proton-ATPase gene, the maximal growth rate is observed exactly at the wild-type level [94]. Other papers from this laboratory describe a similar property for other glycolytic enzymes in *L. lactis*, like LDH, PFK, PK, PGM, PGE, GAPDH, as well as the activity of the entire *las* operon [95-97,67]. For all corresponding genes it was found that maximal growth rate or glycolytic rates were observed at the wild type expression level. Interpreting these results in the framework of metabolic control analysis, the authors concluded that these enzymes have no control on the growth rate or on the glycolytic rate, or technically that the flux control coefficients of the enzymes on these processes equals zero. This is surprising, because in the metabolic control analysis framework the summation theorem for control coefficients says that the sum of control coefficients must be 1. Or in other words, the control must lie in another enzyme, or be distributed over multiple enzymes. However, from an optimization perspective it is easy to understand the results, specifically with respect to the lack of control on growth rate, when growth rate is an entity that determines fitness to a large degree and has been optimized in evolution. The optimal expression of an enzyme, corresponds exactly to the level at which it should have no control over growth rate. This seems to be in conflict with the summation theorem [98], but it is not if we accept that it may be impossible to measure true flux control coefficients in actively regulating systems (see “*In the optimal state apparent in vivo flux control coefficients equal 0*”). The control coefficient of an enzyme is defined as the ratio of relative changes in a flux over relative change in that enzyme, *without changes happening in the other enzymes*. The latter condition can not be guaranteed in living systems, as they may adapt the amounts of other enzymes in response to experimentally induced changes in a target enzyme.

#### In the optimal state apparent in vivo flux control coefficients equal 0

**Figure 2:**
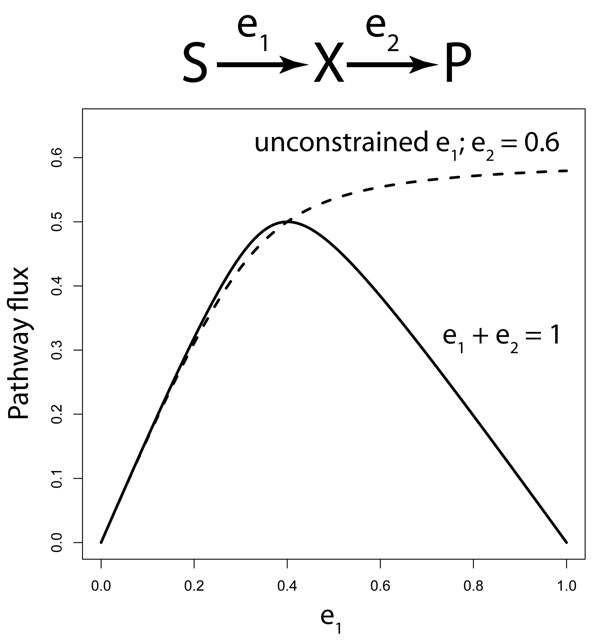
Illustration of the difference between measuring flux in systems without and with regulatory constraints. The flux in a pathway with two irreversible Michaelis-Menten enzymes was calculated. The amount of the first enzyme *e*_1_ was varied either independently of *e*_2_, as it should be to measure its control coefficient, or in a system in which the total amount of enzyme *e*_1_ + *e*_2_ is constrained. In the latter system an optimal distribution of the enzymes is observed at *e*_1_ = 0.4. In the neighborhood of that optimum *e*_1_ has no apparent control on the flux (nor does *e*_2_). Rate equations and parameter values used: *v*_1_ = *k*_1_*e*_1_(*S / K_S_*)/(1 + (*S / K_S_*) + (*X / K_S_K_IX_*))*v*_2_ = *k*_2_*e*_2_*X* / (*K_X_* + *X*) *k*_1_ = 2, *k*_2_ = 1, *K_S_* = 1, *K_IX_* = 5, *K_X_* = 2, and *S* = 5.

The flux control coefficient of an enzyme on a pathway flux is defined as the relative effect of the concentration of the enzyme on the metabolic flux through the pathway. To state this in mathematical terms; suppose we have a pathway with *N* different enzymes *E_i_*, where *i* is an index for the different enzymes running from 1 to *N*. The metabolic flux *J* through the pathway depends on the concentrations *e_i_* of the enzymes *E_i_*, i.e. it is a function of those concentrations. Then the flux control coefficient of *E_i_* is defined as [99]:

If we want to experimentally measure the control coefficient of one of the enzymes on the flux in the pathway, then we could vary the concentration of that enzyme, and measure the resulting changes in the flux. To measure the control coefficient of that enzyme, no changes in the concentrations in the other enzymes are allowed to occur (this is what the partial differentiation ∂*J* / ∂*e_i_* indicates). This is an important condition which is likely not to hold in living systems, as will be discussed below (and illustrated in Figure 2).

The basic problem in optimization is to distribute a limited resource over a number of components so that some function of the components is optimized. In the case of metabolic pathways, the limited resource could be the total amount of protein present in the enzymes. Then adding a little bit of one enzyme would automatically be compensated for by the system by deducing the same total amount of protein from one or more other enzymes. In case of living organisms such limitations could result from the limited space inside cells [84], or the limitation by the total capacity of ribosomes or, assuming that the amount of ribosomes can be adapted, a limitation by multiple physical constraints on the complete self-replicating machinery, which is basically the system of all components in a cell [60]. It is now clear that control coefficients can not be determined in such a cell from observations of the amount of the manipulated enzyme only. When experimentally manipulating the concentration of an enzyme, the cell will automatically compensate this perturbation by changing the concentrations of other enzymes. If we assume that around the enzyme level in the wild type the regulation of enzyme concentrations is optimal, such that the maximal flux is obtained, then any small changes in one enzyme will be compensated exactly by changes in one or more of the other enzymes, leading to apparent control coefficients equal to zero (as also demonstrated in Fig 2).

A simple example can be deduced from a theoretical result by Klipp and Heinrich in [100] where the total amount of protein in a linear pathway was taken as the limiting resource. The authors showed that if that resource is used optimally to attain maximal flux through the pathway, then the flux control coefficients equal the fractional enzyme concentrations, i.e.  where *e_tot_* is the total amount of protein. Rewriting this equation using the definition of , we have in the optimal state, i.e when *J* = *J*_max_:

If the organism is optimally regulating its resource distribution in this state, then any change *δe_k_* in the concentration *e_k_* of an enzyme *E_k_* will be compensated for by a total change –*δe_k_* in the other enzyme concentrations so that  and *e_tot_* remains unchanged. Hence, the net change in flux *δJ* resulting from an engineered change *δe_k_* and induced changes in the other enzymes is by first order approximation

So, although the individual flux control coefficients may not be zero, the effect of optimal regulation, by compensating cost effects of manipulation of a particular enzyme leads to a vanishing net effect *δJ* on the flux *J*. In other words, as a consequence of innate optimal regulation of enzyme expression, the true control coefficients can not be experimentally assessed if only *J* and *e_k_* are measured in the experiment. The apparent control coefficient  will equal zero, even if the true control coefficients differ from zero (Fig 2).

Although this effect is expected to hold only for a flux that is evolutionarily optimized, which in case of growth is the biomass synthesis rate, any flux closely linked to biomass synthesis, like ATP production, or the glycolytic flux, can be expected to behave similarly. So, the seminal work of P.R. Jensen’s group should not be viewed as a failure to figure out where control of glycolysis is located, but as important evidence that *L. lactis* glycolysis has indeed been optimised with respect to enzyme levels. One may conclude that attempts to increase the glycolytic flux, or acidification rate (a combination of flux and growth rate) in *L. lactis* is doomed to fail. The economic perspective to cellular growth strategies, including conceptual models and theory sketched above, allow the test of scenarios in which still more resources may be allocated to glycolysis. These activities could be strengthened by strain diversity studies, in which differences in acidification rates are screened and mapped to molecular mechanisms. Alternatively, one may have to conclude that *L. lactis* has indeed hit the physical boundaries of its biological apparatus, which may be somewhat disappointing, but useful to know. These boundaries are nevertheless likely to be dependent on the environmental conditions, especially if these are dynamic in nutrient composition and competing species. Thus what is optimal in one state is probably sup-optimal in another, and this is still rather uncharted territory in microbial systems biology. In particular, many methods in systems biology, such as FBA, work only for monocultures under constant environments, and there is an urgent need to move to more complex (eco)systems. One theoretical framework that deals with such conditions is evolutionary game theory, which will be illustrated for LAB in the next session.

### Evolutionary game theory of cooperating proteolytic lactococci and other games

*Lactococcus lactis* is one of the dominant bacterial species in many dairy starter cultures [101,102]. Strains of dairy origin have usually several amino acid auxotrophies [103,104] and are therefore dependent on utilizing amino acids present in the growth medium. Bovine milk contains roughly 3% protein, which is mainly present in various forms of casein. The different types of caseins are approximately 200 amino acids in length and have to be cleaved into peptides before they can be taken up and utilized. Lactococci have a sophisticated machinery consisting of an extracellular protease and peptide transport and degradation systems, which allow them to utilize milk protein [105]. The presence of the protease is essential for rapid growth in milk. While lactococcal genomes encode several peptidases and peptide transport systems [106-108], the cell wall anchored protease is usually encoded as a single copy on a plasmid [109-113]. In 1931 Harriman and Hammer [114] first described that starter lactococci that initially grew rapidly in milk lost this ability upon prolonged propagation. They ascribed their observations to the loss of proteolytic activity, but it was not until the 1970s that it was discovered that the protease was encoded on a plasmid that was lost occasionally, giving rise to protease-negative mutants. During prolonged propagation these mutants invade the protease positive population [115]. The fact that the proteolytic trait was highly unstable, while the presence of the protease leads to significantly increased growth rates and biomass yields, seemed counterintuitive, and several studies tried to address this paradoxical behaviour [116,117].

**Figure 3:**
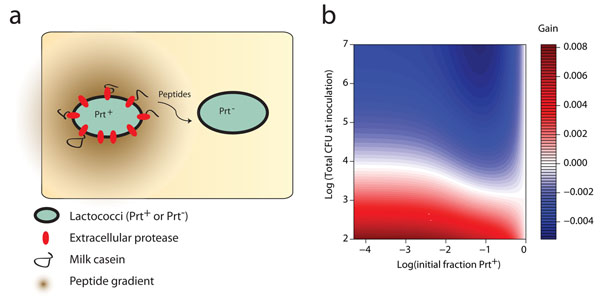
Peptide cross-feeding between Prt^+^ and Prt^-^ strains in a mixed culture of both variants. The extra cellular protease of Prt^+^ strains cleaves casein into peptides that diffuse away from the cell and can be utilized by invading Prt^-^ cheating cells (Panel a). Modelling the dynamics between the two variant strains shows that at low cell densities and low Prt^+^ frequencies the fractional gain after one propagation step is high for Prt^+^ cells. If cultures are grown (inoculated) at high cell desities and high frequencies of Prt^-^ cells the fractional gain of Prt^+^ cells is negative indicating that they are outcompeted by Prt^-^ cells (Panel b). Reproduced from Bachmann *et. al.* 2010 with permission from the publisher.

Given these observations, an obvious question is how such a proteolytic trait can evolve and be stably maintained. Altruistic behaviour on the expression of an extracellular protease has been suggested, based on the observation that protease expression in *Bacillus subtilis* is heterogeneous within a clonal population [118]. Such altruism could play a role in the stabilization of the protease, but it cannot explain its evolution. Only when other system properties, like spatial structure, cell densities and substrate/product diffusion were considered, a model could be developed that explained the observed behaviour (Figure 3) [119]. The model is based on two assumptions. The first assumption is that the expression of the protease imposes a burden on the fitness and growth rate of Prt^+^ relative to Prt^-^ cells. The second assumption is that in milk a Prt^+^ cell can take up a fraction of the extracellular degradation products before they diffuse away. If this is the case in a mixed culture of Prt^+^ and Prt^-^ cells, the burden of protease expression can be compensated by the ability to capture more peptides. This ability increases with decreasing total cell densities and with a decreasing fraction of Prt^+^ cells in the culture (lower left corner in Fig. 3b). At high cell densities and/or high fractions of Prt^+^ cells the peptide concentration in the medium will be sufficient to support high growth rates of cheating Prt^-^ strains, allowing them to invade a Prt^+^ culture (top right corner in Fig. 3b). The validity of this model was confirmed indirectly by propagation experiments of mixed cultures in milk. In a more direct approach the authors showed peptide cross-feeding in a mixed culture through a series of experiments in which bacterial luciferase was coupled to promoter sequences that respond to intracellular amino acid/peptide levels. By varying the Prt^+^: Prt^-^ ratio in mixed cultures and placing the reporter construct in either the Prt^+^ or Prt^-^ strains it was established that at low frequencies of Prt^+^ strains in the culture there is a significant difference in intracellular amino acid/peptide availability between the two variant strains, whereas at high frequencies of Prt^+^ strains no difference could be detected.

Similar concepts are likely to be applicable to other extracellular substrate degrading enzymes. A well-described example is the expression of the sucrose degrading invertase in yeast [120]. Game theoretical approaches showed that with an increasing burden of expressing public goods, population dynamics represent respectively a mutual beneficial relationship, a snowdrift game in which the co-existence of co-operators and cheaters is possible or a prisoners dilemma which leads to the extinction of the co-operators [120,121].

Lactic acid bacteria are relatively well studied with respect to their ability to produce bacteriocins [122,123] and the influence of bacteriocins on bacterial population dynamics has been studied in great detail over the past decades [124-126]. Bacteriocin producing cells and their sensitive as well as resistant derivatives form three interacting strains whose dynamics can also be described by the hand game rock-paper-scissors. In such a game the bacteriocin producer beats the sensitive strain, which beats the resistant strain which beats the producer strain. Mathematical simulations as well as experimental work demonstrated such rock-paper-sccissors dynamics [127] and subsequently it was demonstrated that such dynamics also occur in co-caged mice that were inoculated with either of the three variant strains [128]. In a recent study two *E. coli* strains, each producing a different type of colicin targeting the opponent, were allowed to compete with each other in various environments. The authors found that bacteriocin production at low levels induce bacteriocin production of the opponent, and through this mechanism the strains could defend local niches and co-exist [129]. Analogously to the proposition that bacteriocins rather increase than decrease biodiversity [27,29] it was also proposed that phage-predation promotes diversity [130]. Given this result and the importance of bacteriophages in the dairy industry, their influence on population dynamics and culture stability might have to be reconsidered. One of the future challenges, the description and understanding of bacterial interactions in e.g. multistrain starter cultures, will certainly need to be assisted, and in the ideal case guided, by predictions of mathematical models that should generate testable hypotheses.

### Conclusions and outlook

In this review we have described how systems biology approaches have contributed to the field of microbial physiology, in particular of lactic acid bacteria. Models and theory have been used to provide concise and precise overviews of current knowledge, and to generalise observations into a framework that can provide deeper understanding. Models can also provide explanations of non-intuitive observations, simply because the human mind cannot track many interdependencies, especially if they are highly nonlinear. Systems biology is largely quantitative physiology, and it has become, and will become even stronger in the future, an important approach in biology in general, and LAB microbiology in particular.

In this light, and as promised, a remark on the issue of standardization. Systems biology has clearly pointed at the inefficient and fragmented use of resources within the life sciences. Each lab now uses its own medium, assay buffer conditions and notation of data and model components. Hence data cannot be pooled easily for modelling purposes, or not at all, even if the same question was addressed in the same strain. If we want to become precise and quantitative in biology, this will have to change. For example, we have found substantial physiological differences between *Lactococcus lactis* MG1363 strains used in Dutch laboratories in Groningen, Amsterdam or Ede (NIZO), likely caused by accumulative adaptations to different cultivation histories. For a number of systems biology projects in The Netherlands, we have therefore developed standards for cryopreservation, chemically defined medium and assay buffer (and assays) for all glycolytic enzymes, which has been adopted by the Dutch researchers active in systems biology of *L. lactis*. Although we obviously hope that these standards will be accepted by the community, it is more important that we agree on some standards: the advantages should be obvious. It will also allow better disentanglement of effects caused by external condition and for example genetic diversity.

So what are the further challenges in microbial systems biology? Obviously, we are far away from capturing the complexity of true living cells with current models. Functional genomics tools become more and more quantitative, and provide valuable, comprehensive data sets on relevant processes in the cell. A number of studies from the groups in Toulouse have demonstrated that beautifully for *L. lactis*, in particular the recent study in which protein and mRNA stability were modelled based on transcriptome and proteome data [89,131]. Additional layers of complexity, from the RNA world or from posttranslational modifications, are yet to be disclosed, but the techniques are developing rapidly to do so in a quantitative fashion as well.

Apart from additional components and interactions, we also see a clear trend towards single-cell technologies. At this level, we observe that noise and heterogeneity are crucial factors to be included in our understanding of phenotype [132]. Such stochastic effects, caused by low copy numbers of crucial components (there is only one DNA molecule, or perhaps two [133]!), can drive phenomena of extreme interest in biology but also industrial (food) applications, such as transcriptional burst, cellular decision making into e.g. competence or sporulation in *Bacillus subtilis*[132], bet-hedging strategies, and heterogeneity in survival of stresses. Technologies will rapidly improve that will allow us to quantify more and more properties on a single cell level using microfluidics, quantitative imaging or flow cytometry. These developments will require a different mindset, and concomitant modelling tools that take the stochastic nature of cellular processes into account.

Finally, we need to bridge the gap between cellular processes and population dynamics in communities. Sequencing of such populations and communities has become affordable, giving rise to the field of metagenomics, and early steps into the direction of metatranscriptomics and metametabolomics. Obviously, for LAB and their applications, these developments are extremely relevant. We have discussed how game theoretical approaches can help in understanding general principles and forces at play in populations, giving rise to often non-intuitive phenotype dynamics. At a more molecular level, we have only begun to scratch the surface of compounds involved in the interactions, technologies to measure community fluxes [134], and modelling approaches to accommodate such community dynamics understanding. To illustrate: the well-established tools for genome-scale metabolic modelling only work for homogeneous monocultures. How would one describe the objective of a community? There are only a very few examples in literature that address this issue [135-137]. We are pioneering these type of questions, and the use of genome-scale metabolic models (which are closest to the metagenomic data), in LAB through the yoghurt consortium of *Lactobacillus bulgaricus* and *Streptococcus thermophilus*. We expect major efforts and breakthroughs in the direction of linking microbial physiology with population dynamic modelling and ecological theories.

## List of abbreviations used

CFU: Colony Forming Units; F16bP: Fructose 1,6-bisphosphate; FBA: Flux balance analysis; GAPDH: Glyceraldehyde 3-phosphate dehydrogenase; LAB: Lactic acid bacteria; LDH: Lactate dehydrogenase; PEP: Phosphoenol pyruvate; PFK: Phosphofructokinase; PFL: Pyruvate:formate lyase; PGE: Phosphoglycerate enolase; PGM: Phoshoglycerate mutase; Pi: iIorganic phosphate; PK: Pyruvate kinase; PPP: Pentose phosphate pathway; Prt+/Prt-: Protease positive and negative phenotypes; PTS: Phosphotransferase system.

## Competing interests

The authors declare that they have no competing interests

## Authors' contributions

BT, DM and HB wrote the review together.
